# Supplementary material for: Selective Photothermal Therapy Using Antioxidant Nanoparticles Encapsulating Novel Near-Infrared-Absorbing Platinum(II) Complexes
Source: Nanomaterials (Basel). 2025 May 25;15(11):796. doi: 10.3390/nano15110796 (PMC12158251; doi:10.3390/nano15110796)
Supplement: Supplementary file 1 [file nanomaterials-15-00796-s001.zip › suppinfo.v3.pdf]

Supplementary information

Selective Photothermal Therapy Using Antioxidant Nanoparticles Encapsulating Novel Near-Infrared-Absorbing Platinum(II) Complexes

Ryota Sawamura,[1,*,†] Hiromi Kurokawa,[2,3,†] Atsushi Taninaka,[4,5,†] Takuto Toriumi,[6] Yukio Nagasaki,[7,8] Hidemi Shigekawa,[4] Hirofumi Matsui,[3] and Nobuhiko Iki[1]

[1] Graduate School of Environmental Studies, Tohoku University, 6-6-07 Aramaki-Aoba, Aoba-ku, Sendai, Miyagi 980-8579, Japan
[2] Phycochemy Corporation, X/S worksite, 4-19-1 Midorigahara, Tsukuba, Ibaraki 305-0035, Japan; hkurokawa.tt@md.tsukuba.ac.jp (H.K.)
[3] Faculty of Medicine, University of Tsukuba, 1-1-1 Tennodai, Tsukuba, Ibaraki 305-8575, Japan; hmatsui@md.tsukuba.ac.jp (H.M.)
[4] Institute of Pure and Applied Sciences, University of Tsukuba, 1-1-1 Tennodai, Tsukuba, Ibaraki, 305-8573, Japan; jun_t@bk.tsukuba.ac.jp (A.T.); hidemi@bk.tsukuba.ac.jp (H.S.)
[5] Takano Co. Ltd., 137 Miyada-mura, Kamiina-gun, Nagano 399-4301, Japan
[6] Faculty of Materials for Energy, Shimane University, 1060 Nishikawatsu-cho, Matsue, Shimane 690-8504, Japan; ttoriumi@mat.shimane-u.ac.jp (T.T.)
[7] Graduate School of Pure and Applied Sciences, University of Tsukuba, 1-1-1 Tennodai, Tsukuba, Ibaraki 305-8575, Japan; nagasaki@ims.tsukuba.ac.jp (Y.N.)
[8] Center of Applied Nanomedicine, National Cheng Kung University, No. 35, Xiaodong Rd., Tainan 701, Taiwan
* Correspondence: sawamura@tohoku.ac.jp (R.S.); iki@tohoku.ac.jp (N.I.)
† These authors equally contributed to this work.

Contents

p. S2 Z-contrast images of PtL_2@RNPs (**Figure S1**)

p. S3 Stability of particle size distribution and absorption spectrum of PtL_2@RNPs in a month (**Figure S2**)

Comparison of absorption spectra for PtL_2@RNPs before and after NIR laser irradiation (**Figure S3**)

p. S4 Change in the absorption spectrum of PtL_2@RNPs after five ON/OFF cycle laser irradiation (**Figure S4**)

Influence of high temperature on NIR absorption for PtL_2@RNPs (**Figure S5**)

p. S5 Determination of the photothermal conversion efficiency of PtL_2@RNPs (**Figure S6**)

p. S7 Determination of the photothermal conversion efficiency of PtL_2 (**Figure S7**)

p. S8 Comparison of NIR absorption derived from PtL_2@RNPs in RGK1 cells at different incubation temperatures (**Figure S8**)

Temporal changes in the phase-contrast images of RGM1 and RGK1 cells after NIR laser irradiation (0.28 W, **Figure S9**)

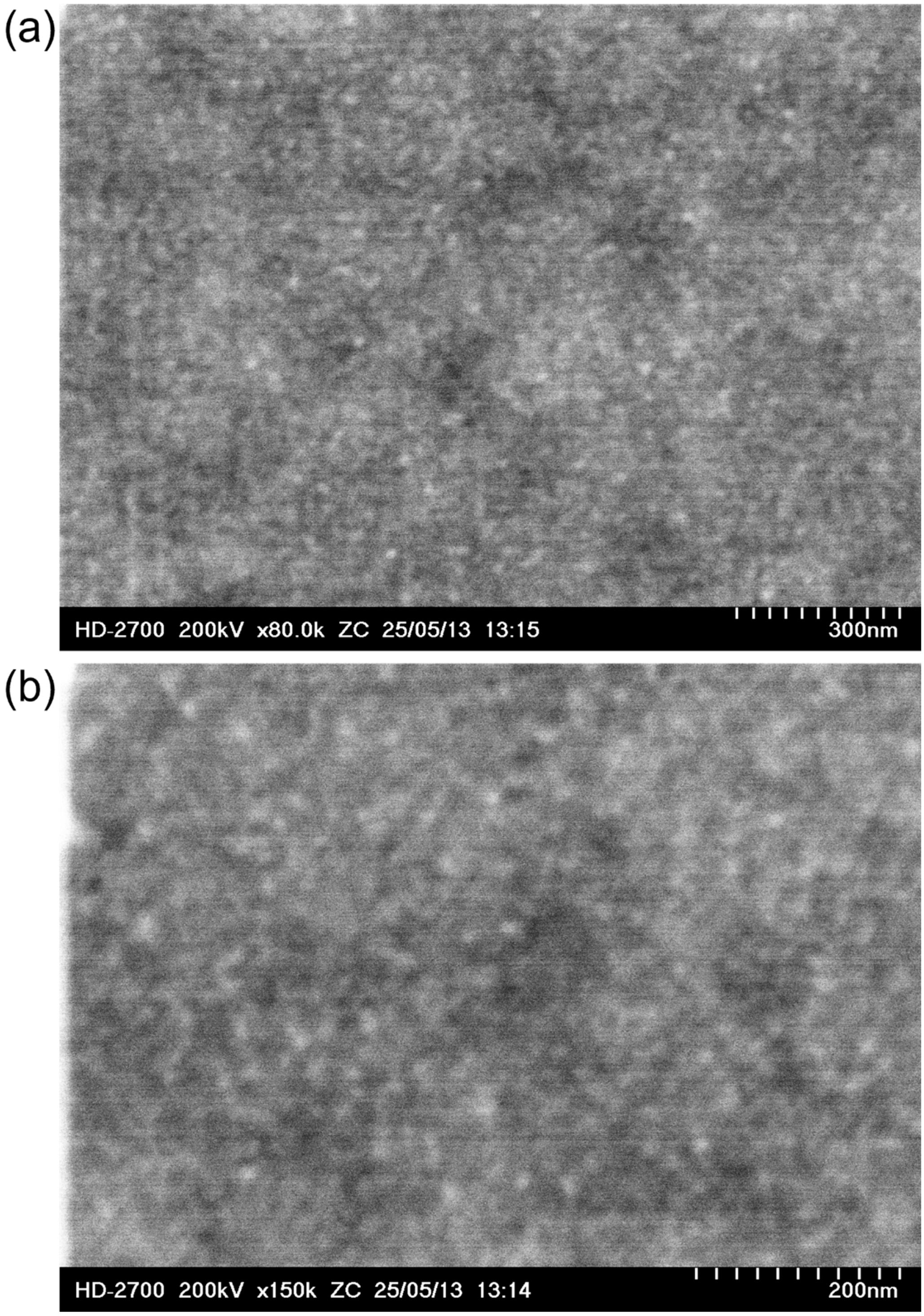

Figure S1 Z-contrast images of PtL_2@RNPs. Scale bars represent (a) 300 nm and (b) 200 nm.

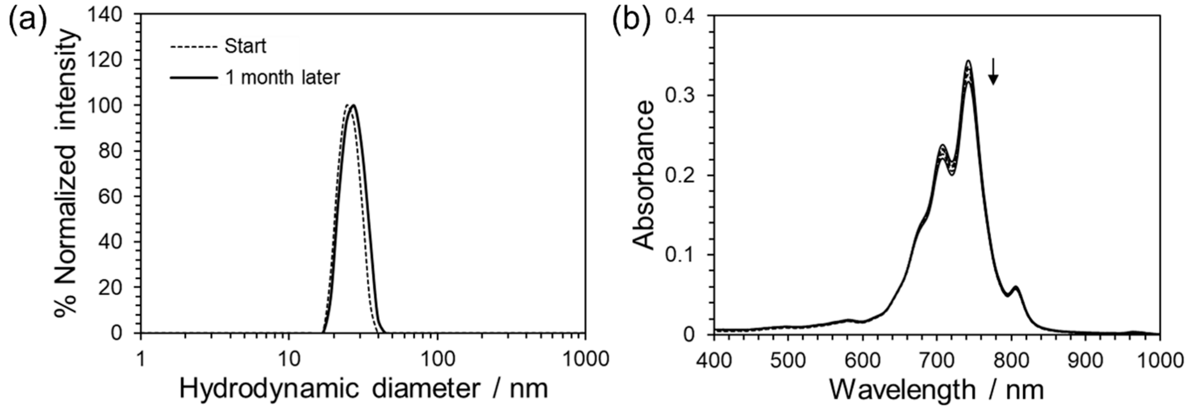

Figure S2 (a) Comparison in the particle size distribution of PtL_2@RNPs in PBS soon (dashed line) and one month (solid line) after preparation. $[Pt^{II}] = 4 \times 10^{-5}$ M. (b) Temporal change of absorption spectra of PtL_2@RNPs in PBS. $[Pt^{II}] = 5.0 \times 10^{-6}$ M. The spectra were measured at 0, 1, 3, 7 days and one month after the preparation.

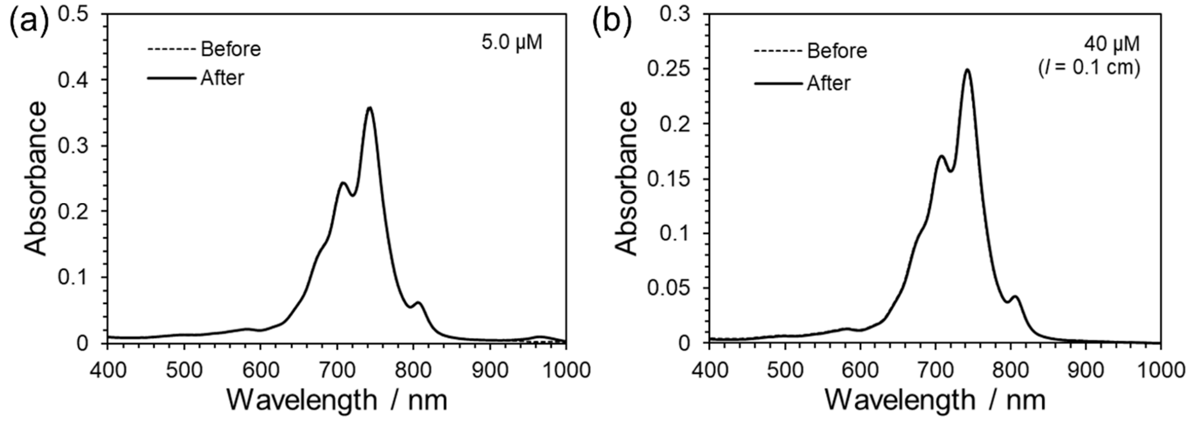

Figure S3 Absorption spectra of PtL_2@RNPs in PBS before and after NIR laser irradiation. $[Pt^{II}]$ = (a) 5.0×10^{-6} M, (b) 4.0×10^{-5} M. The spectrum of the sample at (c) 4.0×10^{-5} M was measured with a light path length of 0.1 cm.

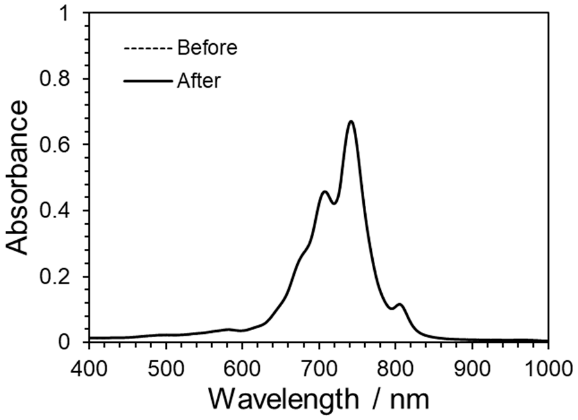

Figure S4 Absorption spectra of PtL_2@RNPs in PBS before and after five-cycle laser irradiation. [Pt^{II}] = 1.0×10^{-5} M.

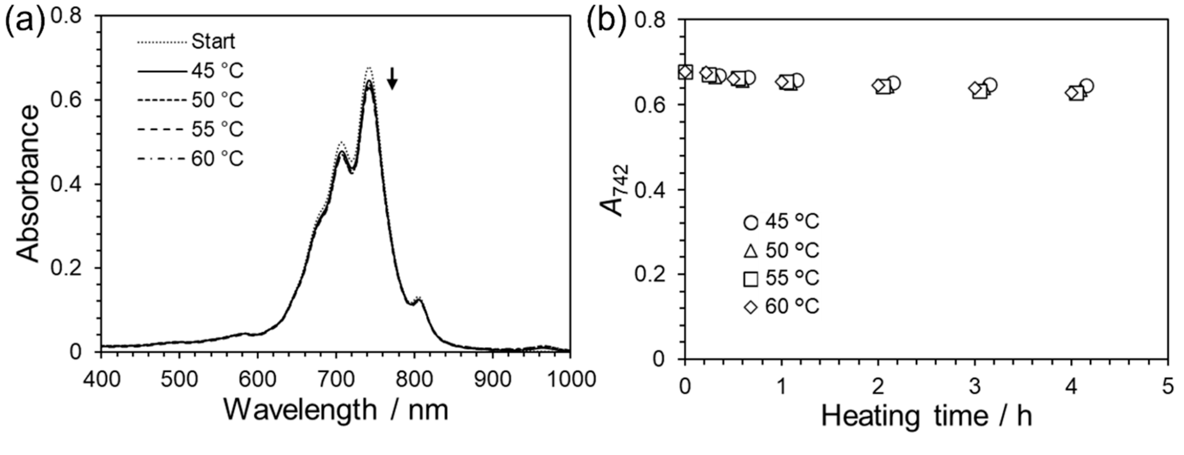

Figure S5 (a) Differences in absorption spectra of PtL_2@RNPs in PBS before ('Start') and after heating at 45, 50, 55, and 60 °C for 4 h. (e) Time course of the absorbances at 742 nm of the PBS suspensions heated at four temperature conditions. [Pt^{II}] = 1.0×10^{-5} M.

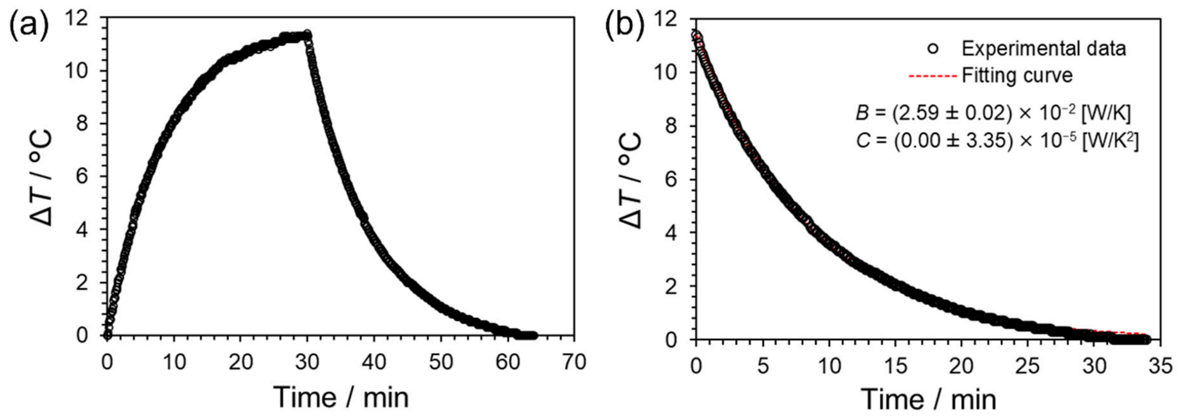

Figure S6 (a) Time course of solution temperature from the initial value (ΔT) of PtL_2@RNPs in PBS irradiated by a 730 nm NIR laser (2 W cm^{-2}) for 30 min following the natural cooling. $[Pt^{II}] = 1.0 \times 10^{-5}$ M. (b) Curve fitting in the cooling period.

The photothermal conversion efficiency of PtL_2@RNPs was calculated based on the reported theoretical model.[S1] The thermal energy balance of the experimental system is described as

$$\left(m_s c_{p,s} + m_c c_{p,c}\right)\frac{d\Delta T}{dt} = Q_{\text{laser}} - Q_{\text{loss}}$$
$$= \{I(1 - 10^{-A_\lambda})\eta + I\xi\} - \{B\Delta T + C(\Delta T)^2\}. \qquad \text{(S1)}$$

The mass of solvent (water, 2 mL) m_s is 2.00 g and the heat capacity of solvent $c_{p,s}$ is 4.19 J g^{-1} K^{-1}. In our system, the mass of cuvette m_c was 5.81 g and the heat capacity of cuvette $c_{p,c}$ was 0.839 J g^{-1} K^{-1}. The ΔT represents the temperature change at time t [s] from the initial temperature (t = 0). The energy given by NIR laser irradiation to the system Q_{laser} [W] is composed of the laser power I [W], the absorbance of PtL_2@RNPs A_λ at the irradiation wavelength λ, the light attenuation rate $1 - 10^{-A\lambda}$, the fraction of light absorbed by the solvent and cuvette ξ, and the photothermal conversion efficiency η. In the reported model, the heat component associated with the photothermal conversion contains the term ($1-\xi$). However, since we measured the absorbance of PtL_2@RNPs using a baseline-corrected spectrometer in a cuvette containing PBS only, we omitted this term. The loss of energy from the system Q_{loss} [W] is described to be a Taylor series of ΔT containing two unknown coefficients B [W K^{-1}] and C [W K^{-2}].

Figure S6a shows the time course of solution temperature from the initial value (ΔT) of PtL_2@RNPs in PBS. The solution is irradiated by the NIR laser until the temperature

S1 H. Chen, L. Shao, T. Ming, Z. Sun, C. Zhao, B. Yang, and J. Wang, *Small*, **2010**, *6*, 2272–2280.

becomes unchanged and then naturally cooled to the initial temperature. The energy balance during the cooling period is expressed to be

$$\left(m_s c_{p,s} + m_c c_{p,c}\right)\frac{d\Delta T}{dt} = 0 - Q_{\text{loss}} = -\{B\Delta T + C(\Delta T)^2\}. \qquad (S2)$$

By solving this differential equation, the following equation in ΔT is obtained.

$$\Delta T = \frac{B\Delta T_0 \exp\left(-\frac{Bt}{m_s c_{p,s} + m_c c_{p,c}}\right)}{B + C\Delta T_0\left\{1 - \exp\left(-\frac{Bt}{m_s c_{p,s} + m_c c_{p,c}}\right)\right\}}. \qquad (S3)$$

The two coefficients B and C are determined by fitting **Equation S3** to the temperature curve during the cooling time using the nonlinear least-square method. We obtained $B = (2.59 \pm 0.02) \times 10^{-2}$ W K^{-1} and $C = (0.00 \pm 3.35) \times 10^{-5}$ W K^{-2} (**Figure S6b**). The average value of C was zero, suggesting that the second-order term in the Taylor series was negligibly small. Furthermore, the ξ value can be calculated from the heat energy balance when the temperature of the solvent only gets constant, as follows,

$$0 = I\xi - \{B\Delta T + C(\Delta T)^2\}$$

$$\xi = \frac{B\Delta T + C(\Delta T)^2}{I}. \qquad (S4)$$

The ξ value in our system was calculated to be $(2.66 \pm 0.02) \times 10^{-2}$. Finally, when the solution temperature becomes constant during the laser irradiation, the energy balance can be written as

$$0 = \{I(1 - 10^{-A_\lambda})\eta + I\xi\} - \{B\Delta T + C(\Delta T)^2\}$$

$$\eta = \frac{B\Delta T + C(\Delta T)^2 - I\xi}{I(1 - 10^{-A_\lambda})}. \qquad (S5)$$

Therefore, the η value of PtL_2@RNPs was determined to be 0.999 ± 0.017 (99.9%).

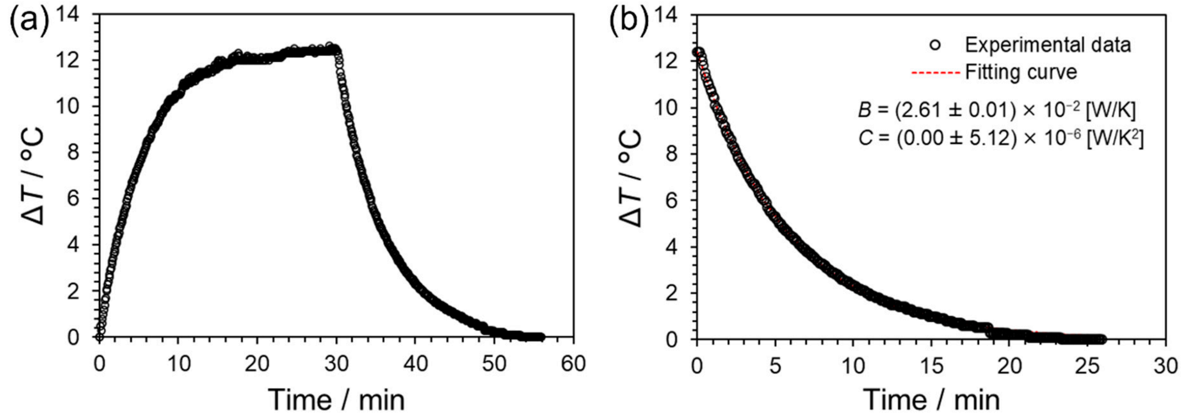

Figure S7 (a) Time course of solution temperature from the initial value (ΔT) of PtL_2 in DMSO irradiated by a 730 nm NIR laser (2 W cm^{-2}) for 30 min following the natural cooling. $[Pt^{II}] = 5.0 \times 10^{-6}$ M. (b) Curve fitting in the cooling period.

The η value of PtL_2 was determined by measuring the solution temperature of PtL_2 in DMSO during the laser irradiation and following natural cooling (**Figure S7a**). The mass of solvent (2 mL) m_s is 2.20 g and the heat capacity of solvent $c_{p,s}$ is 1.97 J g^{-1} K^{-1}. By fitting **Equation S3** to the temperature curve in the cooling period (**Figure S7b**), two unknown coefficients B and C were determined to be $(2.61 \pm 0.01) \times 10^{-2}$ W K^{-1} and $(0.00 \pm 5.12) \times 10^{-6}$ W K^{-2}, respectively. Similar to PtL_2@RNPs, the average value of C was zero. The fraction of light absorbed by the solvent and cuvette ξ was $(7.36 \pm 0.02) \times 10^{-2}$ from the measurement of DMSO only. Finally, the η value of PtL_2 was calculated to be 0.954 ± 0.003 (95.4%).

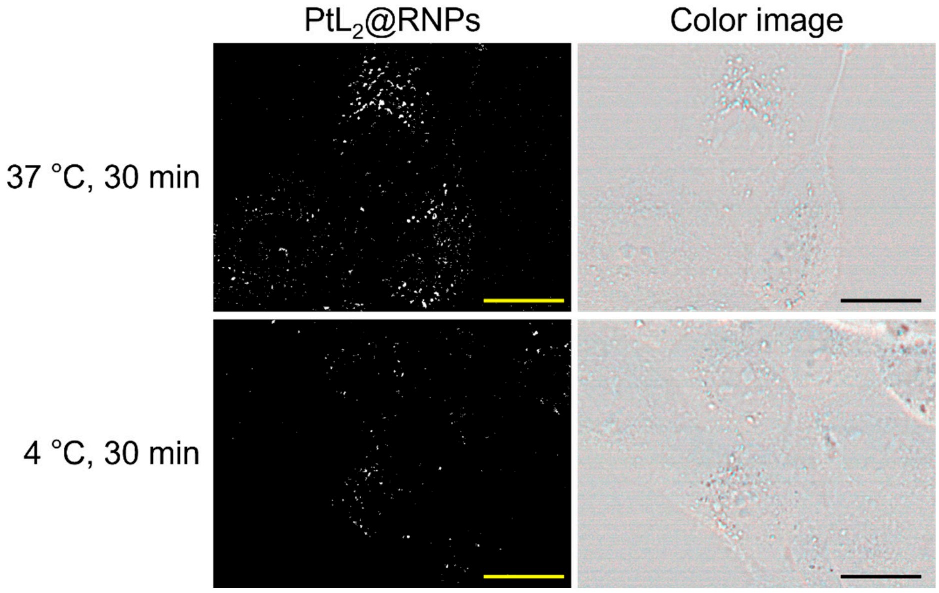

Figure S8 Distributions of NIR absorption derived from PtL_2@RNPs (left) and color images (right) of RGK1 cells incubated with PtL_2@RNPs ($[Pt^{II}] = 4.0 \times 10^{-5}$ M) at 37 °C (upper) or 4 °C (lower) for 30 min. All scale bars represent 10 μm.

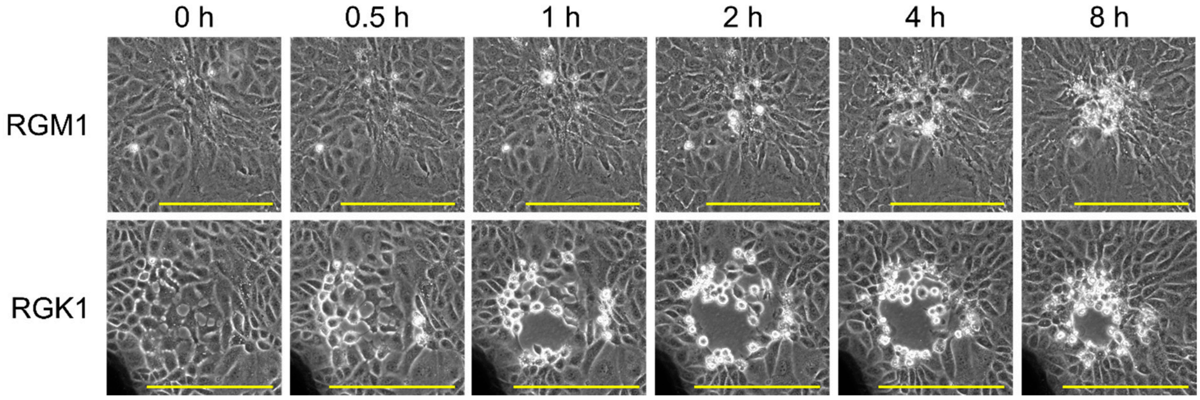

Figure S9 Temporal changes in the phase-contrast images of RGM1 and RGK1 cells containing PtL_2@RNPs after irradiation of 730 nm NIR laser (0.28 W, spot size: ~0.3 mm) for 10 min. All scale bars represent 200 μm.
